# Welcome, 1897

**Published:** 1896-12

**Authors:** 


### EDITORIAL.

### WELCOME, 1897.

In closing the old year with our December number, the Journal welcomes the new year under the brightest and most auspicious skies that have gladdened our efforts for many years. With its future prosperity assured, and its substantial growth during the past year, closing the same with abundant evidences of good-will and help exhibited by our readers, we feel deeply grateful to every contributor from the most eminent to the most humble who has aided us in maintaining and strengthening our publication. The past three years have been the most trying that our profession has had to deal with in the last score of years, and to sustain veterinary journalism and make it broader, stronger, and better has been no light burden upon the shoulders of those who have had the directing of these enterprises. With some the outlay has exceeded the income by hundreds of dollars, and this has been our own experience; but our faith has been strong, and we feel now that the long and trying period of uncertainty has passed, and the measure of support we have received during the past six months is an assurance that our efforts have been appreciated by our readers. We enter upon the new year with a feeling of strength that has not been ours to enjoy before. This fully assures our position in the forefront of the battle for every good and proper aim of the profession. In legislation, in higher education, in aiding control work, in the field of the routine practitioner, in every channel that will aid the advancement of our profession there will be found in 1897, in the foreground of battle, the strong arm of the Journal, sustaining and battling for every good cause for our mutual good, and we shall know no defeat, but will continue the fight until every outpost has been captured and every point of vantage securely placed under our flag. Our increased measure of support by the profession will be returned a hundredfold, and when the evening of 1897 shall close we feel confident and reliant that our advancement in every direction will be of so substantial a character that the arms of the profession will be wide open to welcome to our ranks a larger number of workers than ever before with an increased field of labor for one and all. What we have stood for in the past—a higher ethical standard for our members, striking down of wrongdoers in every direction; fearless and independent in our editorial columns—will be maintained in the coming year, more forceful and potent than ever before. And as increased readers and supporters shall have been added, so wider, broader, and more far-reaching will be our influence in training a keener perception of our needs among a greater number of the profession, and there will surely follow a stronger moral support of every good movement in every community.

We welcome the new year, confident that our own measure of prosperity is only a forecast of the measure of good things to be meted out to our profession, with an assurance of the greater value we can be to them, with the ever-needful reminder that continuous, unceasing effort is the price of success.

